# Toxicity and quality of life report of a phase II study of stereotactic body radiotherapy (SBRT) for low and intermediate risk prostate cancer

**DOI:** 10.1186/s13014-016-0758-8

**Published:** 2017-01-13

**Authors:** Matthew J. Boyer, Michael A. Papagikos, Rex Kiteley, Zeljko Vujaskovic, Jackie Wu, W. Robert Lee

**Affiliations:** 1 Department of Radiation Oncology, Duke University Medical Center, DUMC Box 3085, Durham, NC 27710 USA; 2 Coastal Carolina Radiation Oncology, Wilmington, NC USA; 3 Department of Radiation Oncology, Walter Reed National Military Medical Center, Bethesda, MD USA; 4 Department of Radiation Oncology, University of Maryland School of Medicine, Baltimore, MD USA

**Keywords:** Prostate cancer, Stereotactic body radiation therapy, Toxicity, Quality of life

## Abstract

**Background:**

Clinical data indicates that delivery of larger daily doses of radiation may improve the therapeutic ratio for prostate cancer compared to conventional fractionation. A phase II study of stereotactic body radiotherapy with real-time motion management and daily plan re-optimization for low to intermediate risk prostate cancer was undertaken to evaluate this hypothesis. This report details the toxicity and quality of life following treatment.

**Methods:**

From 2009 to 2013, 60 patients with T1–T2c prostate cancer with a Gleason score of 6 and PSA ≤ 15 or Gleason score of 7 and PSA ≤ 10 were enrolled. Patients with nodal metastases, an American Urological Association symptom score > 18, or gland size > 100 g were not eligible. Patients were treated to 37 Gy in 5 fractions. Early and late genitourinary and gastrointestinal toxicity were graded based on NCI CTCAE v4.0 and quality of life was assessed by the American Urological Association symptom score, International Index of Erectile Function, and Expanded Prostate cancer Index Composite Short Form up to 36 months after treatment.

**Results:**

After a median follow-up of 27.6 months, no grade 3 or greater genitourinary toxicity was observed. Four patients (6.7%) reported a late grade 2 genitourinary toxicity. One patient (1.7%) reported a late grade 3 gastrointestinal toxicity. Five patients (8.3%) developed a late grade 2 gastrointestinal toxicity. The median American Urological Association symptom score increased from 4.5 prior to treatment to 11 while on treatment (*p* < 0.01), but was 5 at 36 months post-treatment (*p* = 0.65). Median International Index of Erectile Function scores decreased from 19 to 17 over the course of follow-up (*p* < 0.01). Only median scores within the Expanded Prostate Cancer Index Composite Short Form sexual domain were significantly decreased at 36 months post-treatment (67.9 vs 45.2, *p* = 0.02). There was no significant difference in median score within the urinary, bowel, or hormonal domains at 36 months of follow-up.

**Conclusions:**

Stereotactic body radiotherapy for low to intermediate risk prostate cancer is well tolerated with limited toxicity or decrease in quality of life. Longer follow-up is necessary to assess the efficacy of treatment.

**Trial registration:**

Clinicaltrials.gov NCT00941915 Registered 17 June 2009.

## Background

Conventional treatment of localized prostate cancer with radiation alone involves doses to 74 Gy or greater given over 8 to 9 weeks. This regimen is based on four randomized trials showing improved progression free survival compared to lower cumulative doses, however at the cost of increased toxicity [1–4]. Given this concomitant increase in toxicity with dose, as well as the expense and inconvenience of protracted courses, alternative treatment schemes have been investigated.

The relationship of cellular death to radiation dose for rapidly dividing cells is dominated by a linear component, represented by a large α/B ratio, such that changes in fraction size have a small impact on efficacy. More slowly dividing cells, with a relatively small α/β are more sensitive to changes in fraction size when delivered to equivalent total doses. There is clinical data [5, 6] that suggests that prostate cancer has a low α/β compared to surrounding normal tissues and therefore increasing daily radiation fraction size will have a greater effect on the tumor and increase the therapeutic ratio. Hypofractionation, or the delivery of fewer, larger fractions to a lower total dose, may allow for increased tumor control and limit the toxicity and practical disadvantages of dose escalation.

Stereotactic body radiation therapy (SBRT) allows for the delivery of large radiation doses through incorporation of coordinate systems and accounting for organ motion *via* daily imaging. Previous reports have demonstrated the feasibility of this technique and high rates of intermediate term biochemical control for patients with low and intermediate risk prostate cancer [7–12]. Here, we report the acute and long-term toxicity of a phase II, multi-institutional study of SBRT for low to intermediate risk prostate cancer.

## Methods

### Patients and eligibility criteria

Men over the age 40 with prostate adenocarcinoma with a Gleason score ≤ 7, clinical stage T1–T2c, and PSA ≤ 15 ng/ml if their Gleason score was ≤ 6 or PSA ≤ 10 ng/ml if their Gleason score was seven were eligible for this study. Other eligibility criteria included a pathological diagnosis within 365 days of enrollment, history and physical exam including digital rectal exam within 8 weeks of enrollment, and a Zubrod performance status of 0–1. Exclusion criteria included node positive or metastatic disease, AUA score > 18, prostate size > 100 g, previous surgery for prostate cancer, prior pelvic irradiation, or previous or concurrent androgen deprivation therapy.

Patients were recruited at three centers: Duke University Medical Center in Durham, NC, Coastal Carolina Radiation Oncology in Wilmington, NC, and Walter Reed National Military Medical Center in Bethesda, MD. Institutional review board approval was obtained at each center and all participants provided written informed consent before registration.

### Treatment planning

The clinical target volume (CTV) was the prostate as determined by planning CT scan with 1 to 1.25 mm slice thickness without contrast. All patients completed a bladder and rectal preparation protocol including 1 tablespoon of milk of magnesia the evenings prior to simulation and treatment and a Fleet’s enema with 16–24 oz of water 2–3 h before simulation and treatments. This was shown to minimize intra-fraction motion based on analysis of the first 15 treated patients. Based on this minimal motion, planning based on fused CT/MRI image sets was allowed after this time. The seminal vesicles were not included in the CTV as the risk of this in patients with a combined Gleason score of seven but primary Gleason score of three has been shown to be 4% [13]. The planning target volume (PTV) was created by expanding the CTV by 5 mm in all directions except by 3 mm posteriorly [9]. The total dose was prescribed to cover ≥ 95% of the PTV *via* intensity modulated radiotherapy (IMRT) with a minimum of seven non-opposed fields or two arc rotational fields. For IMRT, fluence mapping of each field was optimized *via* minimizing a cost function combining PTV coverage and OAR sparing followed by a leaf-motion optimization that converts the fluence map in to a sliding window delivery. And for rotational fields, a similar cost function was used for the optimization, where gantry positions, MLC leaf positions and dose at each gantry position were optimized.

**Fig. 1:**
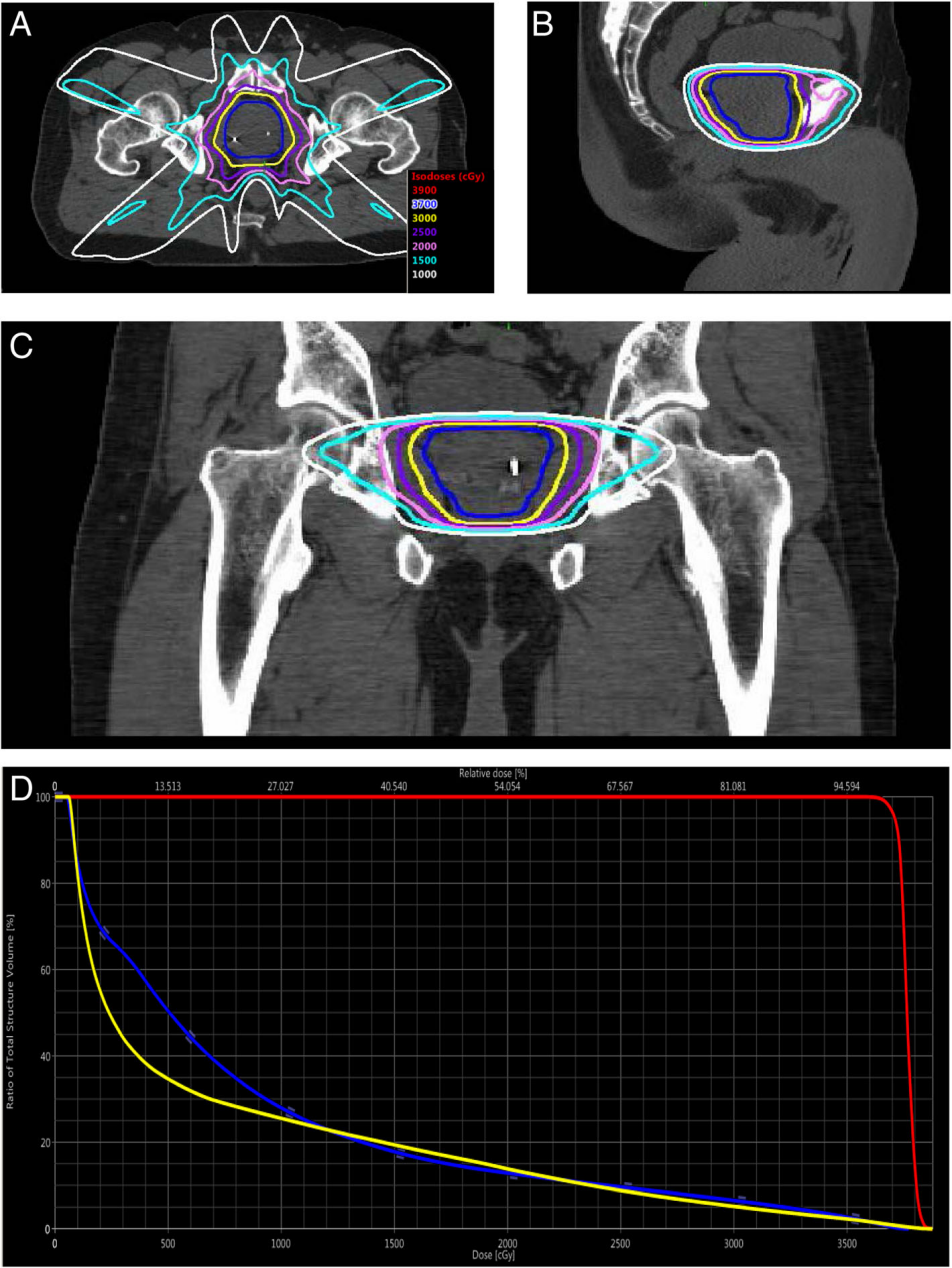
Representative treatment plan. Isodose curves for a representative 7 field treatment plan in the axial (**a**), sagittal (**b**), and coronal (**c**) planes. **d** Dose volume histogram of this plan with PTV in red, rectum in blue, and bladder in yellow

The maximum doses to the rectum and bladder were 105 and 110% of the prescription dose, respectively. Fifty percent of the rectum was limited to ≤ 24 Gy and up to 40 cc of bladder could receive > 24 Gy. The maximum doses to the penile bulb and femoral heads were 100 and 81%, respectively. Isodose curves at mid-prostate for a representative patient are shown in Fig. 1. All plans underwent QA check by principal investigator (WRL) and lead physicist (JW).

### Radiation delivery

Patients were treated SBRT with 6 to 15 MV beams to a total dose of 37 Gy to cover at least 95% of the PTV volume in 5 fractions. Although not based on an external coordinate system, the term SBRT was used in order to be consistent with CPT coding. Treatment machine QA was performed daily and monthly and patient specific QA performed prior to treatment. The dose was selected as an intermediate between among fractionation schedules published at the time of the study design [9–12]. Stereotactic treatment was accomplished with either implanted transponders (Calypso) or ExacTrac system and/or cone beam CT with fiducial markers. 68% of patients were treated using Calypso transponders. Each of the three institutions were required to verify that the margins were sufficient using the technologies in place at each center be it Calypso, ExacTrac or CBCT. Treatment was delivered every other day with a minimum of 36 h to a maximum of 96 h between consecutive treatments. The total duration of treatment was between 10 and 18 days.

### Toxicity and quality of life assessment

Toxicity, as defined by NCI CTCAE v4.0, was assessed at follow-up visits 1, 3, 6, 12, 18, 24, and 36 months following the end of treatment. Acute toxicity was defined as occurring within 90 days of completing treatment. American Urological Association Symptom Scores (AUASS) [14] and International Index of Erectile Function (IIEF) [15] scores were collected once prior to treatment and then following treatment at the above time points *via* questionnaires. Expanded Prostate Cancer Index Composite Short Form (EPIC-26) bowel, urinary, hormonal, and sexual quality of life (QOL) scores [16] were obtained by questionnaire once prior to treatment and at 3, 12, 24, and 36 month follow-up appointments.

### Statistical methods

The primary endpoint of this phase II study was the incidence of acute and late genitourinary (GU) and gastrointestinal (GI) toxicity with the null hypothesis that SBRT is not tolerable. Secondary endpoints included disease-free survival and patient QOL. Estimated grade 3 rectal toxicity for this regimen was 1.5% based on a BED_3Gy_ of 78 Gy in 39 fractions. With 60 patients there would be 77% power to rule out a >7.1% rate of late Grade 3 toxicity. A rate of ≥ 20% of grade 3 or greater GU or GI toxicity was considered unacceptable and 5% acceptable. Interim analyses of the rates of toxicity were planned after 15 and 30 analyzable patients were recruited.

Frequency distributions of patient demographics and grade 0 to 5 GU and GI toxicity were compared using Χ^2^ tests. Actuarial toxicity rates were calculated using the Kaplan-Meier method. QOL scores were normalized to baseline value prior to treatment. The Wilcoxon ranked sign test was used to analyze the change in QOL scores with time. Median follow-up time was computed from the end of treatment to the last follow-up date.

## Results

### Patient demographics

From November 2009 through December of 2013, 60 patients were enrolled. The median age was 66. Median pre-treatment PSA was 5.8. 11 (18%) patients had T2a disease, two (3%) had T2b disease, and the remainder had T1c disease. Twenty- four (40%) patients had a Gleason score of 6 and 36 (60%) had a Gleason score of 7. Twenty (33%) had low risk disease and 40 (67%) had intermediate risk disease. The median follow-up time was 27.6 months (Interquartile range 25–37.0 months). Patient demographics are summarized in Table 1.

**Table 1:** Baseline characteristics of patients

Characteristic	
Age, Median (Range)	66 (49–86)
Race, Number (Percentage)	
White	41 (68.3)
Black	17 (28.3)
Asian	1 (1.7)
Native American	1 (1.7)
PSA, Median (Range)	5.83 (0.5–13.53)
Stage, Number (Percentage)	
T1c	47 (78.3)
T2a	11 (18.3)
T2b	2 (3.3)
Number of Biopsy Cores, Median (Range)	12 (2–26)
Number of Positive Cores, Median (Range)	3 (1–10)
Gleason Score, Number (Percentage)	
6	24 (40)
7	36 (60)
Median Follow-Up, Months	27.6

### GU toxicity

**Fig. 2:**
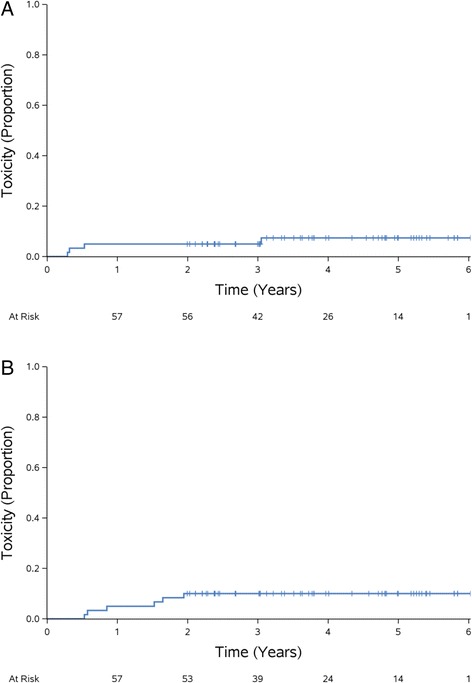
Actuarial rates of toxicity. Actuarial rates of grade 2 or greater late genitourinary (**a**) and gastrointestinal (**b**) toxicity

No grade 3 or greater acute or late GU toxicity was observed (Table 2). Fifteen (25%) patients developed at least one acute grade 2 GU toxicity with 11 of these patients having urinary frequency. Five patients reported grade 2 urinary urgency, three reported grade 2 urinary retention, and two reported grade 2 urinary tract pain. Four (6.7%) patients reported one grade 2 late GU toxicity (hematuria, urinary incontinence, retention, or urgency). The actuarial rate of late grade 2 or greater toxicity was 5.0% (95% CI 0–10.5%) at 2 years (Fig. 2a).

**Table 2:** Crude toxicity rates

Genitourinary Toxicity				Gastrointestinal Toxicity			
Acute		Late		Acute		Late	
Grade	Number (%)	Grade	Number (%)	Grade	Number (%)	Grade	Number (%)
1	44 (73.3)	1	22 (36.7)	1	19 (31.7)	1	15 (25.0)
2	15 (25.0)	2	4 (6.7)	2	3 (5.0)	2	5 (8.3)
3	0 (0)	3	0 (0)	3	0 (0)	3	1 (1.7)
4	0 (0)	4	0 (0)	4	0 (0)	4	0 (0)
5	0 (0)	5	0 (0)	5	0 (0)	5	0 (0)

Rates of early, occurring < 90 days from the end of treatment, and late, occurring >90 days from the end of treatment, toxicity

### GI toxicity

There was one grade 3 late GI toxicity (fecal urgency), otherwise there was no early or late grade 3 or greater GI toxicity (Table 2). Three (5%) patients developed acute grade 2 GI toxicity and 5 (8.3%) patients developed late grade 2 GI toxicity. The most common GI toxicity was rectal hemorrhage. The actuarial rate of late grade 2 or greater toxicity was 10% (95% CI 2.4–17.5%) at 2 years (Fig. 2b).

### Quality of life

**Fig. 3:**
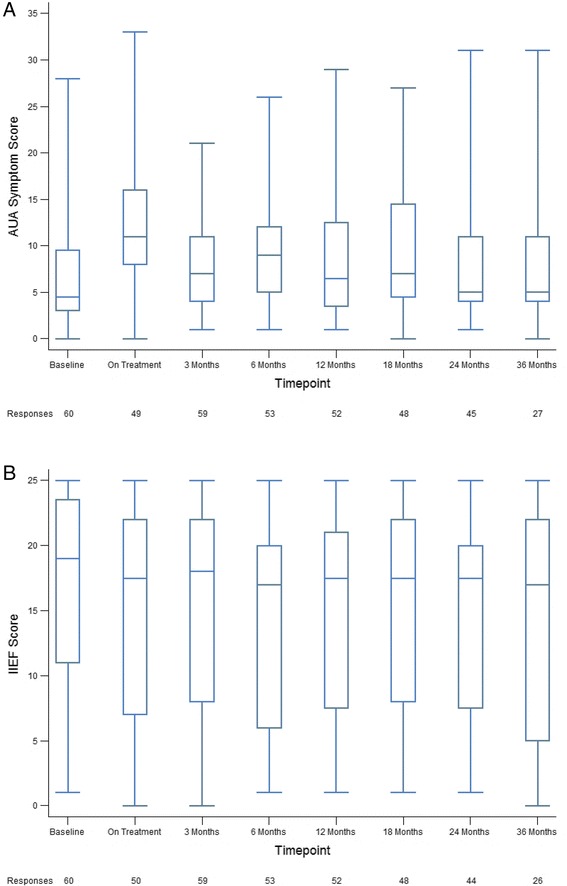
Patient reported QOL scores following treatment. AUASS (**a**) and IIEF (**b**) scores at baseline, on treatment, and 3, 6, 12, 18, 24, and 36 months post-treatment. The number of responses obtained at each time point is indicated

**Fig. 4:**
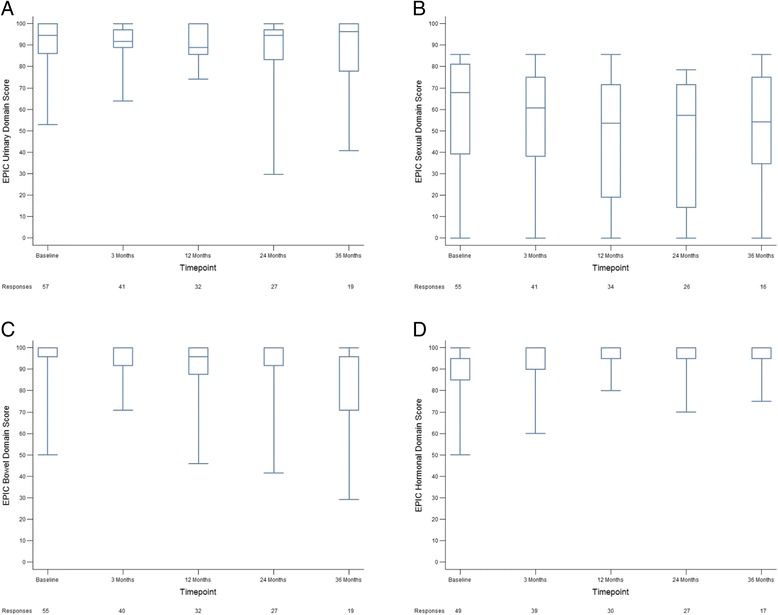
Prostate cancer treatment specific QOL scores following treatment. EPIC-26 scores in the urinary (**a**), sexual (**b**), bowel (**c**), and hormonal (**d**) domains at baseline and 3, 12, 24, and 36 months post-treatment. The number of responses obtained at each time point is indicated

Quality of life up to 36 months from the completion of treatment was assessed by the AUASS, IIEF, and EPIC-26 scores. The median AUASS more than doubled from 4.5 at baseline to 11 (*p* < 0.01) during treatment, however the median AUASS of 5 at 36 months post-treatment approximated the pre-treatment baseline (*p* = 0.65, Fig. 3a). IIEF scores were significantly worse at 3 (median IIEF score 18, *p* = 0.03) and 36 months (median IIEF score 17, *p* = 0.01) post-treatment compared to the pre-treatment baseline however the median score only decreased by 2 from 19 to 17 over this time (Fig. 3b). Consistent with the results of the AUASS, EPIC-26 scores in the urinary domain were significantly decreased from a median baseline of 94.4 pre-treatment to 91.7 at 3 months (*p* < 0.01) and 88.9 at 12 months (*p* < 0.04) following treatment but returned to baseline at later timepoints (median score of 96.3 at 36 months, *p* = 0.65, Fig. 4a). Median EPIC-26 scores in the sexual domain were significantly decreased at 24 (57.1, *p* < 0.01) and 36 (54.2, *p* = 0.02) months post-treatment compared to a pre-treatment median of 67.8 although the number of responses returned at these timepoints were low (Fig. 4b). There was no significant decrease in the 36 month follow-up median EPIC-26 scores in either the bowel (*p* = 0.22) or hormonal (*p* = 0.53) domains. (Fig. 4c-d).

## Discussion

In this report of a phase II multi-institutional study of SBRT for low to intermediate risk prostate cancer low rates of genitourinary and gastrointestinal toxicity were observed with little change in QOL by AUASS as well as IIEF and EPIC-26 scores.

Clinical data [5, 6] suggest that hypofractionated radiation for prostate cancer may improve the therapeutic ratio for this disease. This data has prompted three non-inferiority studies in the last decade investigating treatment in 19 to 28 fractions compared to 39 or more [17–19]. Two of these studies have recently demonstrated hypofractionated treatment to be non-inferior to conventional fractionation with the best estimate hazard ratios for disease free survival favoring hypofractionation [18, 19].

At the same time encouraging results with SBRT for sites outside of the prostate have generated enthusiasm in extending this technique to prostate cancer patients. To this end a number of other phase I and/or II trials have been initiated to demonstrate the feasibility and tolerability of SBRT for prostate cancer.

The observed toxicity of these trials is summarized in Table 3. Consistent with these prior findings, of the 60 patients enrolled on this study only one case of grade 3 gastrointestinal toxicity was observed and no grade 3 or greater genitourinary toxicity was reported. It is possible however that late GU side effects may be underestimated due to the lack of identification of the intraprostatic urethra by imaging or introduction fo a Foley catheter at the time of simulation. There were very similar rates of acute grade 2 GU and GI toxicity (25 and 5%, respectively) in this study as seen in RTOG 04-15 with either hypofractionated (24.7 and 9.7%, respectively) or conventionally fractionated (23.7 and 9.9%, respectively) treatment [18]. Late grade 3 or greater toxicity was rare in this study (1 case of grade 3 GI toxicity) as well as other trials of SBRT (Table 3) or RTOG 04-15 where all rates were 6.6% or less [7–12, 18]. A preliminary report of a randomized phase II trial, RTOG 0938, comparing SBRT in 36.25 Gy in 5 fractions to hypofractionated treatment to 51.6 Gy in 12 fractions for favorable risk prostate cancer showed similar low rates of late grade 3 or greater GI or GU toxicity with SBRT (0.8%) or hypofractionated (1.7%) treatment [20].

**Table 3:** Previously published rates of toxicity following prostate SBRT

Study	n	Dose/Fractions	Timepoint	Genitourinary Toxicity		Gastrointestinal Toxicity	
				Grade 2	≥ Grade 3	Grade 2	≥ Grade 3
Madsen [10]	40	33.5/5	Within or after 1 month	Acute 20.5%	Acute 2.5%	Acute 13%	Acute 0%
				Late 20%	Late 0%	Late 7.5%	Late %
Tang [12]	30	35/5	≤6 months	13%	0%	7%	0%
King [9]	41	36.25/5	≥6 months	24%	5%	15%	0%
McBride [11]	34	37.5–36.25/5	Within or after 3 months	Acute 19%	Acute 0%	Acute 7%	Acute 0%
				Late 17%	Late 2%	Late 7%	Late 5%
Hannan [8]	91	45–50/5	Within or after 9 months	Acute 22%	Acute 0%	Acute 20.9%	Acute 2.2%
				Late 20.9%	Late 5.5%	Late 13.2%	Late 6.6%
Alonghi [7]	40	35/5	Within or after 6 months	Acute 40%	Acute 0%	Acute 10%	Acute 0%
				Late 2.5%	Late 0%	Late 0%	Late 0%

Reported toxicity of previously published phase I and/or II trials of SBRT for prostate cancer; n = number of patients on trial, dose is reported in Gy

Similar to the limited toxicity in this study, follow-up extending out to 36 months post-treatment showed limited to no change in QOL as measured by either AUASS, IIEF scores, or EPIC-26 scores in either the urinary, bowel, sexual, or hormonal domains. There was a significant decline within the bowel domain of the EPIC-26 scores perhaps in concert with the slight increase in late, as compared to early, grade 2 or grade gastrointestinal. Similar increases in late bowel toxicity have been seen in phase III dose escalation studies of conventionally fractionated radiation and hypofractionated versus conventionally fractionated treatment. These results should be interpreted with caution however given the relatively low rate of events in this study and decline in responses to the QOL questionnaires such that less than a third of patients responded to the EPIC-26 questionnaire at 36 months.

It is tempting to comment on the disease status of these patients, however with a short median follow-up of 27.6 months, the ability to make inference on the efficacy of SBRT is limited. Longer follow-up is required for a more robust outcome analysis. If disease control is found to be similar with the observed low rates of toxicity, the option of decreasing treatment time for men with low to intermediate risk prostate cancer will have significant implications for patient convenience and cost.

This study only included men with low to intermediate risk disease and the PSA in those with a Gleason score of 7 was limited to 10 or less. Therefore the results of this study should not be extended to patients with higher risk disease who might require coverage of the seminal vesicles in the treatment volume or those patients treated with concurrent androgen deprivation therapy, both of which were not allowed on this study. In addition it is likely that for at least some men on this study there is little to no advantage to having undergone radiation as compared to active surveillance. The present results should therefore have limited impact on the decision to undergo treatment or not.

## Conclusions

In summary for men with low to intermediate risk prostate cancer toxicity following SBRT is low and similar to conventionally fractionated and hypofractionated treatment with limited to no change in QOL. Further follow-up is warranted to address disease control after this treatment.

## ᅟ

AUASS: American Urological Association Symptom Scores
CTV: Clinical target volume
EPIC 26: Expanded Prostate Cancer Index Composite Short Form
IIEF: International Index of Erectile Function
IMRT: Intensity modulated radiotherapy
PTV: Planning target volume
QOL: Quality of life
SBRT: Stereotactic body radiotherapy
